# Influence of tumour grade on disease survival in male breast cancer patients: a systematic review

**DOI:** 10.1007/s10549-024-07446-z

**Published:** 2024-08-02

**Authors:** Stephen Kinsey-Trotman, Alain Nguyen, Suzanne Edwards, Adam Swalling, Pallave Dasari, David Walsh, Wendy V. Ingman

**Affiliations:** 1 Discipline of Surgical Specialties, Adelaide Medical School, The Queen Elizabeth Hospital, University of Adelaide, 28 Woodville Road DX465702, Woodville South, SA 5011 Australia; 2 Robinson Research Institute, University of Adelaide, Adelaide, SA 5006 Australia; 3 Lyell McEwin Hospital, Northern Adelaide Local Health Network, Haydown Rd, Elizabeth Vale, SA 5112 Australia; 4 School of Public Health, University of Adelaide, Adelaide, SA 5005 Australia; 5 Central Adelaide Local Health Network, 28 Woodville Rd, Woodville South, SA 5011 Australia

**Keywords:** Male breast cancer, Tumour grade, Survival, Prognostic biomarker

## Abstract

**Purpose:**

Histological grading of tumours is a well-established biomarker used to guide treatment in female breast cancer. However, its significance in male breast cancer remains unclear. This systematic review investigates the prognostic significance of tumour grade in relation to breast cancer-specific survival (BCSS) in male breast cancer patients undergoing surgery.

**Methods:**

MEDLINE, PUBMED Central and EMBASE databases were searched to identify randomised trials and observational studies related to male breast neoplasms, tumour grading, recurrence, and survival.

**Results:**

A total of fifteen observational type studies were included in the review. A significant association between tumour grade and BCSS was reported in a majority of studies. This association was most evident with regard to high-grade (grade III) compared to low grade (grade I) tumours, with a significant relationship in 4 out of 4 studies. For intermediate-grade II tumours an association was demonstrated in a minority of studies.

**Conclusions:**

This study confirms an association between high-grade male breast cancers and poorer disease-specific survival, however, the significance of intermediate-grade tumours remains unclear. Further research is required to investigate the biology of male breast cancer in relation to histological grade and optimally define intermediate-grade disease.

**Supplementary Information:**

The online version contains supplementary material available at 10.1007/s10549-024-07446-z.

## Introduction

While prognostic biomarkers provide valuable information to guide optimal treatment in female breast cancer, their utility in male breast cancer is poorly defined [1–3]. One of the earliest independent prognostic indicators in female breast cancer is histological grade, however there are conflicting studies of its significance in male disease. Tumour grade is a histological measure of the invasive potential of the tumour, and reflects the underlying molecular biology of the cancer and it’s immunogenicity [4]. Tumour grade is associated with cell adhesion proteins including P-cadherin, C-terminal tensin-like and claudin 4 [5, 6] and immunomodulatory proteins including Chemokine (CC-motif) ligand 2 and transforming growth factor beta [7, 8] that regulate oncogenesis, differentiation, and cell migration [9].

The current histological grading system used in both female and male breast cancer is the Nottingham Grade System [10]. Grading relies upon the sum of three epithelial cell characteristics which include the degree of structural differentiation as shown by the percentage of tubule formation, the number of mitotic nuclei, and pleomorphism indicated by the degree of nuclear irregularity. Grade I tumours are considered to have a more favourable prognosis. Grade II tumours are intermediate. Grade III tumours are more poorly differentiated and carry a worse prognosis. Although separated into distinct grades, it is important to consider that malignancy occurs on a continuum and refinements to reduce intra- and inter-observer variation and improve the prognostic significance of histological grading in breast and other cancer types is ongoing [11, 12].

An early study by Giordano et al. gave some insights into the prognostic utility of tumour grade in male breast cancer [13]. This study of 2537 male breast cancer patients from US cancer registries suggested that histological tumour grade did not have any significant association with overall survival (OS). In subsequent studies, there have been conflicting reports of the prognostic utility of histological grade to patient survival outcomes in male breast cancer cohorts, although many of these were conducted in small patient numbers [14–17]. Male breast cancer is a rare occurrence, accounting for less than 1% of breast cancer cases [18], and many studies are limited to small sample sizes.

In studies of male breast cancer, patient age is an important consideration as a potential confounder due to the increased risk of cardiovascular and other organ system dysfunction that impacts survival outcomes, as well as reduced tolerability of conventional cancer therapies [19]. Given the advanced age of many male breast cancer patients, survival data may be more instructive when expressed as breast cancer-specific mortality/survival (BCSM/BCSS) rather than overall survival (OS). Other outcome measures commonly reported in trials of female breast cancer patients include disease-free survival (DFS) and distant recurrence-free survival (DRFS). Specifically, the distant recurrence of breast cancer in another organ is a marker of poorer survival outcome [20]. However, both DFS and DRFS are rarely reported outcome measures in male breast cancer cohorts which may be due to both a lack of clinical trial data in this patient group and limitations in outcome data recorded in large observational cancer registries.

A further challenge remains for pathologists in accurately defining tumour grade in male breast cancer specimens where there is a relative paucity of normal breast epithelial cells for comparison. Background non-neoplastic breast tissue in histological specimens is essential for the accurate determination of nuclear pleomorphism, a key element in determining tumour grade [21]. This has led some authors to question the appropriateness of extrapolating female breast cancer grading techniques to the male breast cancer setting [22].

Given these challenges in male breast cancer research, an improved understanding of the significance of histological tumour grade as a prognostic biomarker in male breast cancer is required to optimise care in the clinical setting. This systematic review was conducted to determine if the tumour grade of male breast cancer specimens was associated with BCSS in patients who had undergone surgery.

## Search strategy

This review was performed in accordance with the Preferred Reporting Items for Systematic Reviews and Meta-Analyses (PRISMA) guidelines. The systematic review was dual registered with PROSPERO—protocol number CRD42023456659 (National Institute for Health and Care Research, UK) and Joanna Briggs Institute (University of Adelaide, Australia).

Given the paucity of randomised trials involving male breast cancer patient cohorts, this review permitted the inclusion of observational studies and case-series but excluded review articles, studies of case reports, or where the male breast cancer cohort was less than ten.

A search of the electronic databases MEDLINE, PUBMED Central and EMBASE for relevant published articles was conducted. Search terms included accepted medical subject headings (MeSH, Ovid platform for Medline) or Emtree (EMBASE) headings relevant to the database and clinical area and included terms such as ‘Breast neoplasms, male’, ‘surgery’, ‘Neoplasm, grading’, ‘Neoplasm recurrence, local’, ‘Survival’. These search terms were combined with synonyms developed with the use of a logic grid. These were combined with relevant subcategory headings and free word combination searches such as ‘grade’, ‘grading’, ‘outcome’, ‘death’ and ‘recurrence’ using keyword search function of each database.

Wildcard terms such as mortalit* were used where multiple terms used in the literature share common word-stem. Searching included truncation and Boolean operators between terms.Search terms included accepted medical subject headings (MeSH, Ovid platform for medline) or Emtree (EMBASE) headings relevant to the database and clinical area. Search terms were combined with synonyms developed with the use of a logic grid. Searching included keyword strings, free word combination and wild card terms with truncation and Boolean operators between terms.

Inclusion criteria:

- In English language
- Peer reviewed articles
- Male breast cancer patients where tumour grade and BCSS reported
- No restriction on date range

Exclusion criteria:

- Articles with no English language abstract or main text
- Articles from non-peer reviewed sources
- Articles where BCSS or mortality was not an outcome measure
- Conference abstracts retrieved through electronic databases without supplementary quantitative data available
- Articles where grading is assessed on biopsy material alone
- Studies or case reports where the male breast cancer cohort was less than ten

The review specified inclusion of male breast cancer patients undergoing surgery only, to exclude biopsy only specimens which may be included in studies. This was to circumvent the provisional nature of tumour grading based on biopsy specimens. This subsequent search strategy was modified given some key articles were not mapped to this additional restriction and that the overwhelming majority of abstracts retrieved included surgical specimen information without this added search term.

The literature review was performed by three investigators. Formulation, protocol development and manuscript review was under the supervision of multiple experienced research supervisors. This included a pathologist (AS) experienced in reporting breast pathology specimens who was independent from the main investigating team. Initial scoping review and search strategy was formulated under the advice of an experienced medical librarian. References were uploaded to Covidence program for review. Duplicate references were removed from review. Articles not meeting the inclusion criteria were omitted following duplicate review. Title and abstract screening and subsequent full text review for selected progressed articles was completed in parallel by SKT and AN independently. Conflicts were resolved through review by WI with references included or excluded for progression to full text review or data extraction. Data extraction was performed by SKT with subsequent verification (in series) by AN outside of Covidence program owing to recording limitations. Application of assessment of bias instrument was in duplicate by SKT and AN.

## Assessment of bias instrument

The Newcastle–Ottawa Scale (NOS) was applied to objectively assess the potential for bias for each study considered for data extraction. This was completed by two reviewers with the higher score recorded as the final score. The minimum score set-point for consideration of inclusion for a given study was 5 (out of a possible 9 across three domains). Further, given the potential for wide variation in this result across studies, it was deemed that no greater than 25% variation between the highest scoring study(s) and the lowest scoring study(s) should be accepted. In the event that this range exceeded 25% the lower scoring study(s) would be omitted in sequence until this range reduced to within 25%. For example, this would result in a minimum NOS score of 7 for inclusion where any other study achieved a total of 9.

### Statistical analysis

Statistical analysis was conducted by SE. Articles with report of disease-specific mortality stratified by tumour grade were considered for inclusion in a meta-analysis. Each study was assessed for comparable methods and reported outcomes. Data extraction was undertaken using Microsoft Excel spreadsheet with data imported to Stata (S Release 15.1 College Station, TX: StataCorp LP) statistical software for analysis. Unless otherwise specified, hazards ratios included are from the contributory studies using multivariate analysis in a cox proportional hazards model.

## Results

**Fig. 1:**
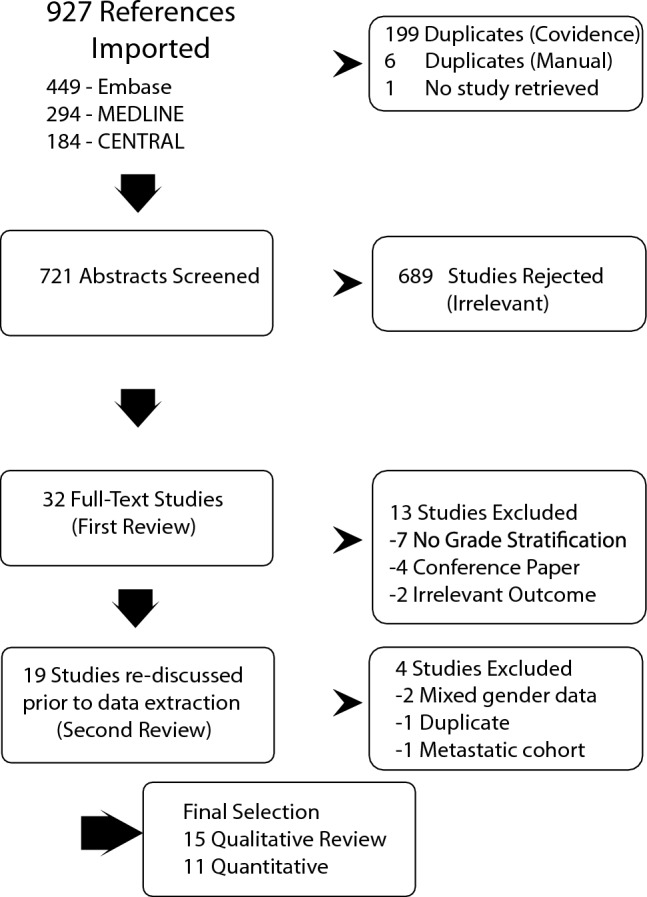
Flow diagram of the PRISMA review process

A total of fifteen observational studies were included for qualitative review (Fig. 1): Macdonald et al. [23]; Cloyd et al. [24]; Nilsson et al. [17]; Madden et al. [25]; Li et al. [26]; Wei et al. [27]; Leone et al. [28]; Pan et al. [29]; Sun et al. [30]; Wang et al. [31]; Han et al. [32]; Cui [33]; Yao et al. [34]; Zhou et al. [35]; Leone et al. [21].

Macdonald et al. [23], Leone et al. [21] and Madden et al. [25] did not report sufficient data for tumour grade in relation to BCSS. The study by Li et al. [26] did not report BCSS data except as a comparison of young male breast cancer patients to females < 40 years of age or males > 40 years of age. These articles were included for qualitative analysis only but did not undergo data extraction (quantitative review) and assessment for bias using Newcastle–Ottawa Scale (NOS) instrument.

Nine out of eleven studies demonstrated a statistically-significant association between grade III tumours and BCSS (reference was grade I tumours except in three studies where grade I/II aggregate was the reference). However, excluding those studies where high-grade tumours were aggregated with undifferentiated tumours, all four remaining studies demonstrated this statistically-significant association. Three out of eight studies demonstrated a significant relationship between grade II versus grade I disease for BCSM (Table 1).

**Table 1:** Breast cancer-specific mortality by tumour grade

Study	BCSM hazard ratio	95% confidence interval	*p*	Cohort
Grade III vs GI/II				
Nilsson et al. [17]	1.5	0.8–2.8	0.16	Swedish registries, *n* = 192
Grade III vs GI				
Cloyd et al. [24]	3.12	2.35–4.65	< 0.05	SEER 1983–2009, *n* = 5425
Han et al. [32]	1.89	1.01–3.54	0.047	SEER 2010–2016, *n* = 3111
Cui [33]	2.56	1.03–6.35	0.043	SEER 1975–2017, *n* = 2099
Yao et al. [34]	2.93	1.04–8.28	0.042	SEER 2010–2014, *n* = 1123
Studies expressing combined analysis of grade III and undifferentiated tumours omitted				
Grade II vs GI				
Cloyd et al. [24]	1.94	1.30–2.90	< 0.05	SEER 1983–2009, *n* = 5425
Wei et al. [27]	2.14	1.30–3.52	0.003	SEER 1990–2010, *n* = 2677
Sun et al. [30]	1.83	1.12–3.00	0.017	SEER 1996–2010, *n* = 1978
Pan et al. [29]	1.58	0.92–2.72	0.096	SEER 1990–2014, *n* = 2713
Han et al. [32]	1.07	0.57–2.02	0.827	SEER 2010–2016, *n* = 3111
Leone et al. [28]	1.071	0.551–2.081	0.840	SEER 2010–2017, *n* = 2389
Cui [33]	1.49	0.61–3.63	0.377	SEER 1975–2017, *n* = 2099
Yao et al. [34]	1.49	0.53–4.24	0.453	SEER 2010–2014, *n* = 1123

The early publication by Macdonald and colleagues gave insight into the outcomes of male breast cancer patients compared to females in a single Canadian province between 1989 and 1998 [23]. Sixty males were compared with 4181 females identified through a British Columbia Breast Cancer Data Registry for disease profile, treatment and outcomes including locoregional relapse, OS and BCSS. This study did not find any significant outcome differences based upon gender. Tumour grade was a significant predictor of locoregional relapse but not survival outcome.

Subsequent studies in North American male breast cancer patients frequently report data retrieved from the Surveillance, Epidemiology and End Results (SEER) program. This database stores demographic, tumour histological data, surgical treatment data as well as survival outcomes. Significantly, data regarding oestrogen and progesterone receptor status was collected from 1990, while HER2 status was reported from 2010. Confounding variables such as socio-economic status, comorbidities, chemotherapy prescription and utilisation of endocrine therapies are lacking. Madden and colleagues study from 2016 gave insight into the treatment landscape of male breast cancer in the United States prior to the multimodality era [25]. Their cohort of 1337 patients were drawn from the SEER registry between the years 1983–2002. While the aim of the study was to determine the impact of adjuvant radiotherapy to survival outcomes, the authors noted higher tumour grade was predictive of poorer overall and cause-specific survival. The larger study by Cloyd et al. investigated the outcomes from breast-conserving surgery compared to mastectomy from the SEER database over the period 1983–2009 [24]. Comprising a total of 5425 patients, the authors noted an increase in lumpectomy over time, rising to 15.1% during the period between 2007 and 2009. For those undergoing lumpectomy, multivariate analysis demonstrated worse outcomes for those with grade II and grade III disease compared with grade I (HR 1.94, 95% CI 1.3–2.90; HR 3.12, 95% CI 2.35–4.65 respectively) over a mean follow-up of 54 months.

Leone and colleagues from Dana Faber Cancer Institute investigated the association of male breast cancer tumour subtype and both disease-specific survival and overall survival through review of SEER data from 2010 to 2017 [28]. Their analysis included data from 2389 males treated for breast cancer with a median follow-up of 43 months (IQR 19–68). There was no significant association found between tumour grade and BCSS for grade II versus I (HR 1.071, 95% CI 0.551–2.081) or grade III/undifferentiated versus grade I (HR 1.834, 95% CI 0.948–3.547). A similar study by Han et al. [32] was published just prior to that of Leone et al., 2021. The study cohort for this project was also drawn from the SEER database between treatment years of 2010–2016, although drawing from a larger male breast cancer cohort of 3111 individuals. These were compared to 404,230 female breast cancer patients. A statistically-significant difference in BCSS was reported between grade III versus grade I disease (HR 1.89, 95% CI 1.01–3.54), although the follow-up period remained undefined. A subsequent study from Leone reported the outcomes of men diagnosed between 1990 and 2008 with stage I–III breast cancer [21]. This research found those patients with higher grade and undifferentiated tumours were at an increased risk of BCSM compared with males with lower grade tumours (HR 1.85, 95% CI 1.22, 2.79).

The study by Li and colleagues compared the survival outcomes of younger male breast cancer patients (under 40 years of age) with both older male breast cancer and female breast cancer SEER cohorts [26]. The younger males were less likely to have lower grade tumours than the older male cohort (42% versus 55%) who had a poorer OS for both grade I/II and grade III tumours (HR 2.66, 95% CI 1.37, 5.20; HR 2.17, 95% CI 1.18, 4.00 respectively).

Studies from Wei et al. [27], Sun et al. [30], Wang et al. [31], Pan et al. [29] and Cui [33] have reported SEER-derived male breast cancer outcomes from extended time periods of study review [27, 29–31, 33]. Wei and colleagues focused on luminal type male breast cancers which were diagnosed between 1990 and 2010 [27]. In the subgroup analysis for tumours with both ER and PR positivity, tumour grade was a significant prognostic indicator for both grade II versus I (HR 2.14, 95% CI 1.30, 3.52) and grade III/IV versus I comparisons (HR 2.77, 95% CI 1.67, 4.60). Another Chinese research team in Nanjing undertook a review of male breast cancer patients treated between 1990 and 2014 with regard to the impact of chemotherapy on survival [29]. This study by Pan et al. reported the outcomes of 2713 male breast cancer patients [19]. High grade disease was associated with a poorer BCSS compared to low grade disease (HR 2.22, 95% CI 1.29, 3.8). Overall, high-grade disease was significantly associated with chemotherapy prescription, although the impact of this treatment upon survival was unclear. Cui published a descriptive paper in 2022 on what is likely the longest period of reported follow-up of male breast cancer patients in the United States [33]. The cohort consisted of national registry patients who were diagnosed between 1975 and 2017. High grade disease was again demonstrated to be associated with poorer breast cancer mortality outcomes compared to low grade disease (HR 2.56, 95% CI 1.03, 6.35). Similar studies were conducted by Sun et al. and Wang et al. during the above period, each with fifteen years of patient study-years [30, 31]. The former demonstrated an association of poorer BCSM for both high-grade and moderate grade disease compared to low grade disease (HR 2.13, 95% CI 1.29, 3.51; HR 1.83, 95% CI 1.12, 3.00 respectively). The analysis by Wang et al. included a female comparator cohort and reported comparable results in BCSM risk for stratification by grade).

Two further research centres published studies in 2022 that utilised much narrower durations of interest from the SEER database. These articles by Yao et al. and Zhou et al. reaffirmed the association of high-grade disease with poorer BCSS [34, 35]. The former also analysed the association between grade II versus grade I for both male patients and a female comparator cohort with a non-significant association found in the male cohort (HR 1.49, 95% CI 0.53, 4.24).

The study by Nilsson et al. utilised both Swedish National Cancer Register and hospital data to report outcomes for patients treated between 1990 and 2005 [17]. This study specifically aimed to review the prognostic impact of histological features of individual male breast cancers and the reclassification of tumour specimens based upon Nottingham Grade criteria using central pathology review is a particular strength of the study. A total of 197 patients from two regions of Sweden were included in the study with a mean follow-up of 54 months (0–180). Univariate analysis comparing grade I, II versus grade III tumours did not demonstrate any difference in BCSM (HR 1.5, 95% CI 0.8–2.8).

Following application of the Newcastle–Ottawa Scale all eleven studies considered for data extraction remained for quantitative analysis. All studies met the minimum total score threshold (total score ≥ 5) with a result range of ≤ 25%.

The planned meta-analysis was not completed as the maximum number of studies that could be included in each pooled analysis was two. This was due to the majority of the included studies utilising data from shared data registries with overlapping years of patient follow-up, raising the possibility of common patient data across studies.

## Discussion

This systematic review of the utility of tumour grade as a prognostic biomarker in male breast cancer is believed to be the only dedicated review of its type. It supports an association between high tumour grade disease and poorer disease-specific survival in a male population. What is less clear is the prognostic significance of intermediate tumour grade, where there was limited association with BCSS when compared to low grade. Tumour grade is a reflection of the morphological, immunohistochemical and molecular features of the cancer; if intermediate-grade does not exhibit increased risk of mortality compared to low grade, it’s biological and prognostic significance becomes questionable.

The biological mechanisms that underpin malignancy in male breast cancer are poorly understood. Given the relationship between tumour grade and BCSS, it is possible that many of the cellular mechanisms of invasion in female breast cancer occur in male disease. However, the lack of a clear association between intermediate-grade and prognosis suggests there may be some sex-specific differences as well. Although tumour size and lymph node status are accepted, independent prognostic biomarkers in male breast cancer [17, 36], the prevalence of lymph node positivity is higher in small (< 20 mm) male breast cancers compared to female disease suggesting differences in tumorigenesis, differentiation and cell migration. Interestingly, there are differences in BCSS in males and females with high (> 30) Oncotype DX 21-gene assay recurrence scores; female breast cancer patients with high risk disease do significantly better than their male counterparts [37, 38]. While the reason for this is unclear, it may relate to differences in adjuvant endocrine therapy use in male patients [39, 40], or an underlying difference in tumour biology. Estrogen receptor-positive cancers may display different proliferation profiles in males compared to females due to differences in circulating sex hormones and this could affect the number of mitotic nuclei. It is suggested that the mitotic count is the most important aspect of tumour grade that drives it’s prognostic significance in female breast cancer [12], and this cell characteristic may require a different cut off to define intermediate-grade in male disease.

A possible contributing factor to the uncertain prognostic significance of intermediate-grade disease is the high proportion of grade II tumours reported in most male breast cancer datasets including the studies comprising this review. Tumour grading in female breast cancer has benefited from several significant contributory studies aimed at improving the reproducibility of results [12]. It remains unclear to what degree, if any, the grading of male breast cancers has benefited from these advancements. With limitations including the relative lack of normal epithelial cells for accurate determination of pleomorphism, there is a high level of discordance between pathologists in male breast cancer tumour grade, particularly in classifying grade II tumours [41]. Given the difficulties with assessing tumour grade in male breast specimens combined with the rarity of the malignancy, the accurate grading in male cancers presents a unique challenge to the pathologist [22, 42].

The studies included in the review encompass a broad period of patient follow-up between the years 1975 and 2017. In a rare malignancy such as male breast cancer, longer periods of interest permit for larger patient numbers leading to potentially higher overall statistical power in studies. However, this does lead to several potential limitations. Studies that include patient groups prior to the 1990s reflect the older Scarff-Bloom-Richardson grading system which has some minor variations from the current Nottingham Grading System. Also noteworthy is the infrequent attribution of grade IV to undifferentiated tumours in a number of studies which is not endorsed by either grading system. Further, the evolution of adjuvant therapies has progressed considerably over the above time period yet the impact upon patient outcomes remains difficult to determine owing to limited recording of adjuvant treatments in national databases such as SEER.

A recurring limitation in studies of male breast cancer patients is that of modest patient numbers. In this current review the opportunity for pooled analysis of patient data remained enticing but was limited by potential overlap between studies. Given only two studies were from populations outside of the United States, the potential for individual patient data to be shared across studies was high. This meant that a meta-analysis could realistically only include limited studies in each data aggregate. As the number of studies was too small for pooled analysis, it was concluded that a meta-analysis would not be appropriate.

A significant limitation of our review is the lack of high-quality randomised trials in the literature for male breast cancer. The included studies are observational in nature and would often be best described as ‘descriptive’. Such non-randomised studies may be impacted by various forms of bias. Complicating this further, no single instrument to assess bias in observational studies is considered the ‘gold standard’. The review utilised the Newcastle–Ottawa Scale owing in part to its simplicity. Alternative instruments such as Robins I, Robins E and EPHPP (Effective Public Healthcare Panacea Project) questionnaires are also available but require further training to be used effectively. Particular consideration must be made to survival data obtained from observational datasets. A number of included studies utilised SEER registry data which is limited by the accuracy of cause of death recording which may be derived from death certificates. In particular, the distinction between those patients succumbing to breast cancer and others dying with their disease by other comorbidities may not be overt. Further, the reliance upon these data registries limits analysis of other instructive outcome measures such as disease-free survival.

This review confirms an association between high-grade male breast cancers and poorer disease-related survival. The prognostic significance of intermediate-grade cancers in male populations remains uncertain. Further research is required to investigate the biology of male breast cancer in relation to histological grade and optimally define intermediate-grade disease.

## Supplementary Information

Below is the link to the electronic supplementary material.

Supplementary file1 (DOCX 15 KB)

## Data Availability

Data available on request.

## References

[1] Bloom HJ, Richardson WW (1957) Histological grading and prognosis in breast cancer; a study of 1409 cases of which 359 have been followed for 15 years. Br J Cancer 11(3):359–377 13499785 10.1038/bjc.1957.43 PMC2073885

[2] Kollias J, Murphy CA, Elston CW, Ellis IO, Robertson JF, Blamey RW (1999) The prognosis of small primary breast cancers. Eur J Cancer 35(6):908–912 10533470 10.1016/s0959-8049(99)00056-8

[3] Smith JA 3rd, Gamez-Araujo JJ, Gallager HS, White EC, McBride CM (1977) Carcinoma of the breast: analysis of total lymph node involvement versus level of metastasis. Cancer 39(2):527–532 837335 10.1002/1097-0142(197702)39:2<527::aid-cncr2820390221>3.0.co;2-n

[4] Takahashi H, Oshi M, Asaoka M, Yan L, Endo I, Takabe K (2020) Molecular biological features of nottingham histological grade 3 breast cancers. Ann Surg Oncol 27(11):4475–4485 32436191 10.1245/s10434-020-08608-1 PMC7808708

[5] Qi R, Lin J, Chen S, Jiang J, Zhang X, Yao B et al (2022) Breast cancer prognosis and P-cadherin expression: systematic review and study-level meta-analysis. BMJ Support Palliat Care 12(e6):e893–e905 32943470 10.1136/bmjspcare-2020-002204

[6] Lanigan F, McKiernan E, Brennan DJ, Hegarty S, Millikan RC, McBryan J et al (2009) Increased claudin-4 expression is associated with poor prognosis and high tumour grade in breast cancer. Int J Cancer 124(9):2088–2097 19142967 10.1002/ijc.24159

[7] Chavey C, Bibeau F, Gourgou-Bourgade S, Burlinchon S, Boissiere F, Laune D et al (2007) Oestrogen receptor negative breast cancers exhibit high cytokine content. Breast Cancer Res 9(1):R15 17261184 10.1186/bcr1648 PMC1851386

[8] Figueroa JD, Flanders KC, Garcia-Closas M, Anderson WF, Yang XR, Matsuno RK et al (2010) Expression of TGF-beta signaling factors in invasive breast cancers: relationships with age at diagnosis and tumor characteristics. Breast Cancer Res Treat 121(3):727–735 19937272 10.1007/s10549-009-0590-z PMC4159718

[9] McSherry EA, Donatello S, Hopkins AM, McDonnell S (2007) Molecular basis of invasion in breast cancer. Cell Mol Life Sci 64(24):3201–3218 17957337 10.1007/s00018-007-7388-0 PMC11136013

[10] Elston CW, Ellis IO (1991) Pathological prognostic factors in breast cancer. I. The value of histological grade in breast cancer: experience from a large study with long-term follow-up. Histopathology 19(5):403–10 1757079 10.1111/j.1365-2559.1991.tb00229.x

[11] Samaratunga H, Egevad L, Yaxley J, Perry-Keene J, Le Fevre I, Kench J et al (2024) Gleason score 3+3=6 prostatic adenocarcinoma is not benign and the current debate is unhelpful to clinicians and patients. Pathology 56(1):33–38 38071161 10.1016/j.pathol.2023.10.005

[12] van Dooijeweert C, van Diest PJ, Ellis IO (2022) Grading of invasive breast carcinoma: the way forward. Virchows Arch 480(1):33–43 34196797 10.1007/s00428-021-03141-2 PMC8983621

[13] Giordano SH, Cohen DS, Buzdar AU, Perkins G, Hortobagyi GN (2004) Breast carcinoma in men: a population-based study. Cancer 101(1):51–57 15221988 10.1002/cncr.20312

[14] Kornegoor R, Verschuur-Maes AH, Buerger H, Hogenes MC, de Bruin PC, Oudejans JJ et al (2012) Immunophenotyping of male breast cancer. Histopathology 61(6):1145–1155 22958056 10.1111/j.1365-2559.2012.04330.x

[15] Lautrup MD, Thorup SS, Jensen V, Bokmand S, Haugaard K, Hoejris I et al (2018) Male breast cancer: a nation-wide population-based comparison with female breast cancer. Acta Oncol 57(5):613–621 29276849 10.1080/0284186X.2017.1418088

[16] Jylling AMB, Jensen V, Lelkaitis G, Christiansen P, Nielsen SS, Lautrup MD (2020) Male breast cancer: clinicopathological characterization of a National Danish cohort 1980–2009. Breast Cancer 27(4):683–695 32108307 10.1007/s12282-020-01066-3 PMC7297815

[17] Nilsson C, Johansson I, Ahlin C, Thorstenson S, Amini RM, Holmqvist M et al (2013) Molecular subtyping of male breast cancer using alternative definitions and its prognostic impact. Acta Oncol 52(1):102–109 22928693 10.3109/0284186X.2012.711952

[18] Bhardwaj PV, Gupta S, Elyash A, Teplinsky E (2024) Male breast cancer: a review on diagnosis, treatment, and survivorship. Curr Oncol Rep. 10.1007/s11912-023-01489-z 38224426 10.1007/s11912-023-01489-z

[19] Flannery MA, Culakova E, Canin BE, Peppone L, Ramsdale E, Mohile SG (2021) Understanding treatment tolerability in older adults with cancer. J Clin Oncol 39(19):2150–2163 34043433 10.1200/JCO.21.00195 PMC8238902

[20] Solomayer EF, Diel IJ, Meyberg GC, Gollan C, Bastert G (2000) Metastatic breast cancer: clinical course, prognosis and therapy related to the first site of metastasis. Breast Cancer Res Treat 59(3):271–278 10832597 10.1023/a:1006308619659

[21] Leone J, Hassett MJ, Freedman R, Tolaney S, Graham N, Tayob N et al (2023) Mortality risks over 20 years in men with stage I-III hormone receptor-positive breast cancer. Cancer Res. 10.1158/1538-7445.SABCS22-PD6-08 10.1001/jamaoncol.2023.7194 PMC10905378 38421673

[22] Johansson I, Killander F, Linderholm B, Hedenfalk I (2014) Molecular profiling of male breast cancer—lost in translation? Int J Biochem Cell Biol 53:526–535 24842109 10.1016/j.biocel.2014.05.007

[23] Macdonald G, Paltiel C, Olivotto IA, Tyldesley S (2005) A comparative analysis of radiotherapy use and patient outcome in males and females with breast cancer. Ann Oncol 16(9):1442–1448 15972730 10.1093/annonc/mdi274

[24] Cloyd JM, Hernandez-Boussard T, Wapnir IL (2013) Outcomes of partial mastectomy in male breast cancer patients: analysis of SEER, 1983–2009. Ann Surg Oncol 20(5):1545–1550 23460016 10.1245/s10434-013-2918-5

[25] Madden NA, Macdonald OK, Call JA, Schomas DA, Lee CM, Patel S (2016) Radiotherapy and male breast cancer: a population-based registry analysis. Am J Clin Oncol 39(5):458–462 24781343 10.1097/COC.0000000000000078

[26] Li N, Wang X, Zhang H, Wang H (2018) Young male breast cancer, a small crowd, the survival, and prognosis? A population-based study. Medicine (Baltimore) 97(40):e12686 30290658 10.1097/MD.0000000000012686 PMC6200462

[27] Wei JL, Zhang JX, Fu DY (2018) Characterization and prognosis of estrogen receptor-positive/progesterone receptor-negative male breast cancer: a population-based study. World J Surg Oncol 16(1):236 30558615 10.1186/s12957-018-1539-7 PMC6297954

[28] Leone J, Freedman RA, Lin NU, Tolaney SM, Vallejo CT, Leone BA et al (2021) Tumor subtypes and survival in male breast cancer. Breast Cancer Res Treat 188(3):695–702 33770314 10.1007/s10549-021-06182-y

[29] Pan H, Zhang K, Wang M, Ling L, Wang S, Zhou W (2020) The effect of chemotherapy on survival in patients with nonmetastatic male breast cancer: a population-based observational study. Cancer 126(Suppl 16):3830–3836 32710661 10.1002/cncr.32829

[30] Sun W, Cheng M, Zhou H, Huang W, Qiu Z (2019) Nomogram predicting cause-specific mortality in nonmetastatic male breast cancer: a competing risk analysis. J Cancer 10(3):583–593 30719155 10.7150/jca.28991 PMC6360428

[31] Wang Y, Chen K, Yang Y, Tan L, Chen L, Zhu L et al (2019) Incidence and survival outcomes of early male breast cancer: a population-based comparison with early female breast cancer. Ann Transl Med 7(20):536 31807518 10.21037/atm.2019.10.04 PMC6861739

[32] Han Y, Wang J, Wang Z, Xu B (2021) Sex-based heterogeneity in the clinicopathological characteristics and prognosis of breast cancer: a population-based analysis. Front Oncol 11:642450 33718239 10.3389/fonc.2021.642450 PMC7945032

[33] Cui X (2022) The prevalence and death risk of male breast cancer: a study based on the surveillance, epidemiology, and end results database. Am J Mens Health 16(1):15579883221074818 35094596 10.1177/15579883221074818 PMC8808035

[34] Yao N, Shi W, Liu T, Siyin ST, Wang W, Duan N et al (2022) Clinicopathologic characteristics and prognosis for male breast cancer compared to female breast cancer. Sci Rep 12(1):220 34997151 10.1038/s41598-021-04342-0 PMC8741943

[35] Zhou Q, Zhang Q, Zhao S, Zhang Y, Wang Q, Li J (2022) A novel nomogram for predicting breast cancer-specific survival in male patients. Am J Clin Oncol 45(10):427–437 36106711 10.1097/COC.0000000000000943

[36] Cutuli B, Le-Nir CC, Serin D, Kirova Y, Gaci Z, Lemanski C et al (2010) Male breast cancer. Evolution of treatment and prognostic factors. Analysis of 489 cases. Crit Rev Oncol Hematol 73(3):246–254 19442535 10.1016/j.critrevonc.2009.04.002

[37] Massarweh SA, Sledge GW, Miller DP, McCullough D, Petkov VI, Shak S (2018) Molecular characterization and mortality from breast cancer in men. J Clin Oncol 36(14):1396–1404 29584547 10.1200/JCO.2017.76.8861 PMC6075854

[38] Grenader T, Yerushalmi R, Tokar M, Fried G, Kaufman B, Peretz T et al (2014) The 21-gene recurrence score assay (Oncotype DX) in estrogen receptor-positive male breast cancer: experience in an Israeli cohort. Oncology 87(1):1–6 24970679 10.1159/000360793

[39] Eggemann H, Ignatov A, Smith BJ, Altmann U, von Minckwitz G, Rohl FW et al (2013) Adjuvant therapy with tamoxifen compared to aromatase inhibitors for 257 male breast cancer patients. Breast Cancer Res Treat 137(2):465–470 23224235 10.1007/s10549-012-2355-3

[40] Anelli TF, Anelli A, Tran KN, Lebwohl DE, Borgen PI (1994) Tamoxifen administration is associated with a high rate of treatment-limiting symptoms in male breast cancer patients. Cancer 74(1):74–77 8004585 10.1002/1097-0142(19940701)74:1<74::aid-cncr2820740113>3.0.co;2-#

[41] O’Malley FP, Pinder SE, Mulligan AM (2011) Breast pathology. Elsevier/Saunders, Amsterdam

[42] Vermeulen MA, Slaets L, Cardoso F, Giordano SH, Tryfonidis K, van Diest PJ et al (2017) Pathological characterisation of male breast cancer: results of the EORTC 10085/TBCRC/BIG/NABCG international male breast cancer program. Eur J Cancer 82:219–227 28292559 10.1016/j.ejca.2017.01.034

