# Supplementary material for: Influence of tumour grade on disease survival in male breast cancer patients: a systematic review
Source: Breast Cancer Res Treat. 2024 Aug 2;208(1):1–8. doi: 10.1007/s10549-024-07446-z (PMC11452471; doi:10.1007/s10549-024-07446-z)
Supplement: Supplementary file 1 — Supplementary file1 (DOCX 15 KB) [file 10549_2024_7446_MOESM1_ESM.docx]

# Supplementary

## Search Strategy

A search of the electronic databases ‘MEDLINE’ and ‘PUBMED Central’ and ‘EMBASE’ for relevant published articles was undertaken in May 2023. Search terms included accepted medical subject headings (MeSH, Ovid platform for Medline) or Emtree (EMBASE) headings relevant to the database and clinical area and included terms such as ‘Breast neoplasms, male’, ‘surgery’, ‘Neoplasm, grading’, ‘Neoplasm recurrence, local’, ‘Survival’. These search terms were combined with synonyms developed with the use of a logic grid. These included keyword strings such as ‘breast cancer’, ‘breast neoplasm’ and ‘breast carcinoma’ with restriction on studies to male/men.

These were combined with relevant subcategory headings and free word combination searches such as ‘grade’, ‘grading’, ‘outcome’, ‘death’ and ‘recurrence’ using keyword search function of each database.

Wildcard terms such as mortalit* were used where multiple terms used in the literature share common word-stem. Searching included truncation and Boolean operators between terms.

## Adjustment of Search Strategy

The initial search strategy which utilised the additional search term ‘surgery’ failed to return a key paper found in earlier scoping review. As a result the search strategy was modified by deleting this search term in favour of a less restrictive review.

The search strategy returned 294 abstracts using Medline (Ovid), 449 using EMBASE and 184 using Central databases. Initially 205 duplicate abstracts were removed from review. This resulted in 721 abstracts for initial review. Following dual review of 721 abstracts, 80 discrepancies existed between reviewers and required resolution with third reviewer. A total of 32 articles proceeded to full text review. This resulted in 20 studies being forwarded for review prior to data extraction with no further discrepancies between reviewers. Upon review of these studies short-listed for data extraction it was noted that two further papers be recommended for removal as either duplicate to or significantly similar to other publications. Two studies from Wu et al were also not eligible for data extraction owing to data reporting aggregated hazards ratios with female patients. The article by Zhou et al 2022 reported survival data for 207 male breast cancer patients with bone metastasis was excluded as was deemed a too restrictive patient subgroup. Four publications were included for qualitative review but were unable to be considered for data extraction owing to reporting limitations.

## Medline Search Strategy

Database: Ovid MEDLINE(R) ALL <1946 to August 04, 2023> Search Strategy: 1 Breast Neoplasms, Male/ (3375) 2 ((male or men) adj10 (breast cancer* or breast neoplasm* or breast carcinoma*)).ti,ab,kf. (3796) 3 1 or 2 (5670) 4 Neoplasm Grading/ (26021) 5 (grade* or grading).ti,ab,kf. (563033) 6 4 or 5 (573152) 7 Neoplasm Recurrence, Local/ (144455) 8 exp treatment outcome/ (1247552) 9 Outcome Assessment, Health Care/ (82177) 10 Survival/ (4923) 11 (survival or death* or mortalit* or outcome* or recurrence* or disease free).ti,ab,kf. (4618005) 12 7 or 8 or 9 or 10 or 11 (5270164) 13 3 and 6 and 12 (294)

## Meta-Analysis

The study by Nilsson et al was the only study on male breast cancer patients outside the United States. The remaining studies invariably reported outcomes of patients with data extracted from the SEER database which placed additional limitations on meta-analysis inclusions. Previous authors have indicated a watershed date of 2010 for SEER database entries of breast cancer patient data with newer entries containing more detailed receptor status information, specifically tumour HER2 status. For a number of studies this date was noted to be either the end point or start point for patient accrual. Given the potential for considerable patient overlap between American studies drawing upon the same data registry, no two studies were combined in pooled analysis for any year of diagnosis except where accrual began or ended in 2010. The studies by Pan et al 2020 and Cui et al 2022 reported outcomes from patients diagnosed over a wide study period and were not able to be included in any pooled analysis owing to patient overlap with every other US study. Further, the study by Wei et al 2018 was limited by report of hazards ratio for a progesterone positive subgroup only and was not included in the meta-analysis.
